# Novel genetic variants of *KHDC3L* and other members of the subcortical maternal complex associated with Beckwith–Wiedemann syndrome or Pseudohypoparathyroidism 1B and multi-locus imprinting disturbances

**DOI:** 10.1186/s13148-022-01292-w

**Published:** 2022-05-28

**Authors:** Laura Pignata, Francesco Cecere, Ankit Verma, Bruno Hay Mele, Maria Monticelli, Basilia Acurzio, Carlo Giaccari, Angela Sparago, Jose Ramon Hernandez Mora, Ana Monteagudo-Sánchez, Manel Esteller, Arrate Pereda, Jair Tenorio-Castano, Orazio Palumbo, Massimo Carella, Paolo Prontera, Carmelo Piscopo, Maria Accadia, Pablo Lapunzina, Maria Vittoria Cubellis, Guiomar Perez de Nanclares, David Monk, Andrea Riccio, Flavia Cerrato

**Affiliations:** 1 Department of Environmental Biological and Pharmaceutical Sciences and Technologies (DiSTABiF), Università Degli Studi Della Campania “Luigi Vanvitelli”, Caserta, Italy; 2 Institute of Genetics and Biophysics (IGB), “Adriano Buzzati-Traverso”, Consiglio Nazionale Delle Ricerche (CNR), Naples, Italy; 3 Department of Biology, Università Degli Studi Di Napoli “Federico II”, Naples, Italy; 4 Cancer Epigenetic and Biology Program (PEBC), Imprinting and Cancer Group, Institut d’Investigació Biomedica de Bellvitge (IDIBELL), Avinguda Granvia, L’Hospitalet de Llobregat, Barcelona, Spain; 5 Josep Carreras Leukeamia Research Institute, Can Ruti, Cami de les Escoles, Badalona, Barcelona, Spain; 6 Centro de Investigacion Biomedica en Red Cancer (CIBERONC), Madrid, Spain; 7 Institucio Catalana de Recerca I Estudis Avançats (ICREA), Barcelona, Catalonia Spain; 8 Physiological Sciences Department, School of Medicine and Health Sciences, University of Barcelona (UB), Barcelona, Catalonia Spain; 9 Molecular (Epi)Genetics Laboratory, Rare Diseases Research Group, Bioaraba Health Research Institute, Araba University Hospital-Txagorritxu, C/Jose Atxotegi s/n, 01009 Vitoria-Gasteiz, Spain; 10 CIBERER, Centro de Investigación Biomédica en Red de Enfermedades Raras, Madrid, Spain; 11 Institute of Medical and Molecular Genetics, INGEMM-Idipaz, Madrid, Spain; 12 ITHACA, European Reference Network, Brussels, Belgium; 13 Division of Medical Genetics, Fondazione IRCCS “Casa Sollievo Della Sofferenza”, 71013 San Giovanni Rotondo, FG Italy; 14 Medical Genetics Unit, University and Hospital of Perugia, Perugia, Italy; 15 Medical and Laboratory Genetics Unit, “Antonio Cardarelli” Hospital, 80131 Naples, Italy; 16 Medical Genetics Service, Hospital “Cardinale G. Panico”, 73039 Tricase, Lecce Italy; 17 School of Biological Sciences, University of East Anglia, Norwich, NR4 7TG UK

**Keywords:** Multi-locus imprinting disturbance, Beckwith–Wiedemann syndrome, Pseudohypoparathyroidism, Genomic imprinting, DNA methylation, Maternal-effect variants, Subcortical maternal complex, Recurrent pregnancy loss, Infertility

## Abstract

**Background:**

Beckwith–Wiedemann syndrome (BWS) and Pseudohypoparathyroidism type 1B (PHP1B) are imprinting disorders (ID) caused by deregulation of the imprinted gene clusters located at 11p15.5 and 20q13.32, respectively. In both of these diseases a subset of the patients is affected by multi-locus imprinting disturbances (MLID). In several families, MLID is associated with damaging variants of maternal-effect genes encoding protein components of the subcortical maternal complex (SCMC). However, frequency, penetrance and recurrence risks of these variants are still undefined. In this study, we screened two cohorts of BWS patients and one cohort of PHP1B patients for the presence of MLID, and analysed the positive cases for the presence of maternal variants in the SCMC genes by whole exome-sequencing and in silico functional studies.

**Results:**

We identified 10 new cases of MLID associated with the clinical features of either BWS or PHP1B, in which segregate 13 maternal putatively damaging missense variants of the SCMC genes. The affected genes also included *KHDC3L* that has not been associated with MLID to date. Moreover, we highlight the possible relevance of relatively common variants in the aetiology of MLID.

**Conclusion:**

Our data further add to the list of the SCMC components and maternal variants that are involved in MLID, as well as of the associated clinical phenotypes. Also, we propose that in addition to rare variants, common variants may play a role in the aetiology of MLID and imprinting disorders by exerting an additive effect in combination with rarer putatively damaging variants. These findings provide useful information for the molecular diagnosis and recurrence risk evaluation of MLID-associated IDs in genetic counselling.

**Supplementary Information:**

The online version contains supplementary material available at 10.1186/s13148-022-01292-w.

## Background

Imprinting disorders (IDs) are a group of human diseases affecting growth, metabolism and neuro-behavioural functions that are caused by deregulation of genes with monoallelic and parent-of-origin dependent expression, known as imprinted genes. Most imprinted genes are organized in clusters in which gene expression is regulated by genomic regions showing differential DNA methylation (Differentially methylated regions, DMRs) between the two parental alleles. Loss of differential methylation of the DMRs is a hallmark of most imprinting disorders [1]. Methylation abnormalities can affect a single or multiple DMRs (multi-locus imprinting disturbances, MLID) and can be associated with genetic variants acting *in cis* or *in trans*.

Beckwith–Wiedemann syndrome (BWS, OMIM #130,650, prevalence of 10,500 live births) is a human condition that is part of a clinical spectrum (BWSp) characterized by more specific or “cardinal” features (e.g. macroglossia, exomphalos, lateralized overgrowth) and less specific or “suggestive” features (e.g. neonatal macrosomia, facial naevus flammeus, polyhydramnios, ear creases or pits, abdominal wall defects). The imprinted gene cluster associated with BWS is located on chromosome 11p15.5 and is organized in two domains that are controlled by the *H19/ IGF2*:IG-DMR (also known as IC1) and *KCNQ1OT1*:TSS-DMR (also known as IC2), respectively. Gain of IC1 methylation and loss of IC2 methylation are found in 5–10% and 50% of the patients with BWSp, respectively. In addition, uniparental paternal disomy (UPD) of chromosome 11p15.5 (upd(11)pat) is reported in 20% of the cases, single nucleotide variants (SNVs) causing loss of function of *CDKN1C* in 5% and copy number variants (CNVs) of chromosome 11p15.5 in 1–2%. About one third of the patients with loss of IC2 methylation display MLID [2].

Pseudohypoparathyroidism (PHP) is a heterogeneous group of endocrine disorders characterized by renal resistance to parathyroid hormone (PTH), causing hypocalcaemia, hyperphosphataemia and elevated circulating PTH levels. PHP is also characterized by several other clinical features such as brachydactyly, short stature, stocky build, round face and subcutaneous ossification, also described as Albright hereditary osteodystrophy (AHO) with or without obesity. PHP is associated with pathogenic variants and/or methylation defects within the imprinted *GNAS* cluster on 20q13.32 [3]. Among the subtypes of PHP, PHP1B (OMIM#603,233) (or iPPSD3, according to the current nomenclature) is clinically characterized by isolated renal PTH resistance, in some cases by thyroid-stimulating hormone (TSH) resistance, and very rarely by some features of AHO. All patients with PHP1B have methylation defects at the *GNAS A/B* maternally methylated DMR, and possibly aberrations at other DMRs in the *GNAS* locus. These methylation abnormalities are supposed to result in decreased expression of the GNAS-Gs transcript [4].

Approximately, 15% of the PHP1B cases are inherited and caused by microdeletions affecting the adjacent *STX16* locus and causing loss of methylation of the *GNAS A/B*:TSS-DMR [5]. Within the sporadic cases, characterized by methylation defects encompassing all the four DMRs of the locus, 10–25% are associated with complete or segmental upd(20)pat [5], while the rest are considered to present primary epimutations. MLID has been detected in a subset of the cases with methylation abnormalities of the whole *GNAS* locus, although its incidence is still undefined due to limited investigation [6–9].

The molecular mechanism causing MLID in BWS and PHP1B is unknown. Its clinical consequences are uncertain although atypical phenotypes or phenotypes overlapping multiple imprinting disorders have been reported in several cases [10–13]. An accurate prevalence of MLID has not been determined yet because there is still no international consensus for definition of MLID and recommendations for accurate molecular testing (number and genomic loci to analyse, and tissue to analyse). Rare loss-of-function variants, affecting genes encoding either zygotic or oocytes-specific trans-acting factors, have been associated with various imprinting disorders and MLID. In particular, zygotic biallelic variants of the *ZFP57* gene have been found in case of Transient Neonatal Diabetes Mellitus (TNDM) [14], while biallelic or heterozygous variants affecting some of the genes encoding the subcortical maternal complex (SCMC) proteins have been identified in the healthy mothers of children affected by BWS or SRS [13, 15–19]. Interestingly, while MLID associated with *ZFP57* variants consistently affect a limited number of imprinted loci, number and methylation levels of the imprinted loci affected by SCMC variants are variable. Moreover, SCMC variants appear to be associated with increased risk of recurrence and reproductive problems, making their identification important for genetic counselling. However, due to the limited number of studies performed so far, further research is needed to identify all the genes involved in MLID and their clinical consequences.

The SCMC is composed of a group of proteins expressed in the oocytes and preimplantation embryos of mammals. This complex plays a crucial role in oocyte to embryo transition carrying out multiple biological functions in the initial stages of embryogenesis, including meiotic spindle formation and positioning, regulation of translation, organelle redistribution, and epigenetic reprogramming [20]. The proteins NLRP5, KHDC3L, TLE6 and OOEP have been identified as members of the human SCMC through protein–protein interaction studies [21]. Other proteins (e.g. NLRP2, NLRP4, NLRP7, PADI6 and ZBED3) have been proposed to be part of the complex on the basis of their co-localization or shared biological functions with other SCMC components, or because they are orthologues of the mouse SCMC members [22–26].

In addition to imprinting disorders, SCMC variants have been associated with severe clinical conditions affecting reproduction such as female infertility, recurrent biparental hydatidiform mole (RHM) and recurrent miscarriages [20, 27]. However, the role of the individual SCMC components in specific clinical conditions and biological processes is not well defined yet. For instance, *KHDC3L* and *TLE6* variants have been associated only with severe non-viable conditions, such as RHM and zygotic lethality, so far [28–31]. Differently, variants of *NLRP7*, *PADI6*, *NLRP2* and *NLRP5* have been associated with both severe reproductive problems and imprinting disorders [17, 18, 32, 33].

Here, we report the results of genetic/epigenetic analyses performed on an Italian and a Spanish cohort of patients with MLID and the clinical features of either BWSp or PHP1B. We have identified maternal putatively damaging variants of several SCMC genes in ten pedigrees, including seven patients clinically affected by BWSp and three by PHP1B. The affected genes also included *KHDC3L* that has not been associated with MLID so far. Our findings further add to the list of the SCMC components and maternal variants that are involved in MLID, as well as of the associated clinical phenotypes.

## Results

### Identification of MLID and DMR methylation profile

The patients 1–7 described in the present study received a clinical diagnosis of BWS. Three of them derived from an Italian cohort of fifteen BWS patients with MLID, the other four from a Spanish cohort of nine patients. Patients 8–10 received a clinical diagnosis of PHP1B. They derived from a Spanish cohort of thirteen PHP1B patients with MLID. The clinical features of the ten patients are summarized in Table 1. Further details and the clinical history of their families are reported in Additional file 1: Families Information and Additional file 2: Fig. S1.

**Table 1:** Summary of the clinical and molecular features of the probands and their families

Family	Proband sex and age (years)	Maternal effect variant	Genotype	Hypomethylated and hypermethylated loci	Maternal reproductive history	Family history of note	Clinical features of proband
1	Female, 24	*KHDC3L* novel NM_001017361: c.296C > G; p.Thr99Arg AF: - GF: - PolyPhen-2: Possibly damaging SIFT: Deleterious SDM: Destabilizing	M: hom P: het F: wt	Hypomethylated loci: *KCNQ1OT1,* *ZDBF2/GPR1,* *NAP1L5,PLAGL1,* *GRB10, PEG10, MEST, ZNF597, GNAS-XLas, GNAS A/B, GNAS-AS1* Hypermethylated loci: *DIRAS3:TSS, GNAS-NESP, ZNF331-DMR1, ZNF331-DMR2*	None	None	Macroglossia, macrosomia, umbilical hernias, ear creases/pits, nevus flammeus (eyelid), mild neonatal hypoglycaemia, pre-eclampsia, prognathism, maxillary hypoplasia, seizures (once), strabismus BWS score: 7
2	Female, 39	*PADI6* novel NM_207421: c.356 T > C; p.Leu119Pro AF: - GF: - PolyPhen-2: Probably damaging SIFT: Deleterious SDM: Destabilizing	M: het P: het S: het F: wt	Hypomethylated loci: *KCNQ1OT1, PPIEL, NAP1L5,* *PLAGL1, ZNF331-DMR1, ZNF331-DMR2, GNAS-XLas, GNAS A/B, GNAS-AS1* Hypermethylated loci: *ZNF597,* *GNAS-NESP*	Two miscarriages	Second sister: three miscarriages and one healthy daughter Third sister: two healthy children	Macroglossia, polyhydramnios, diastasis recti, neonatal hypoglycaemia, ear creases/pits, nevus flammeus, nephromegaly, enlarged pancreas, facies grossolana, face asymmetry, maxillary hypoplasia, mild intellectual deficit, psychomotor retardation BWS score: 8
3	Female, 40	*NLRP5* rs768443657 NM_153447.4: c.2615G > A; p.Arg872Lys AF: 8.95E-06 GF: 0 PolyPhen-2: Possibly damaging SIFT: Deleterious SDM: Destabilizing *NLRP5* rs36118060 NM_153447.4: c.3584G > A; p.Arg1195Gln AF: 0.145 GF: 0.022 PolyPhen-2: Benign SIFT: Tolerated SDM: Destabilizing *NLRP4* rs111284755 NM_134444.4: c.1279G > A; p.Ala427Thr AF: 0.006 GF: 2.00E-05 PolyPhen-2: Benign SIFT: Tolerated SDM: Destabilizing	M: het P: wt F: na M: het P: het F: na M: het P: het F: na	Hypomethylated loci: *KCNQ1OT1, DIRAS3:TSS, PLAGL1, GRB10,* *ERLIN2, GNAS-XLas, GNAS A/B, GNAS-AS1* Hypermethylated loci: *ZNF331-DMR1,ZNF331-DMR2, GNAS-NESP*	Ovarian stimulation Two miscarriages. One triplet pregnancy: one miscarriage, two children born at 6 months of gestation and died few hours/days later. One healthy son	Proband: one healthy daughter one miscarriage, one healthy son	Macroglossia, macrosomia, lower limbs dysmetria, scoliosis, hypotonia BWS score: 5
4	Male, 15	*NLRP5* rs34175666 NM_153447.4: c.1685G > A; p.Arg562His AF: 0.007 GF: 0 PolyPhen-2: Possibly damaging SIFT: Deleterious SDM: Destabilizing *NLRP5* rs12462795 NM_153447.4: c.3323C > G; p.Ser1108Cys AF: 0.145 GF: 0.022 PolyPhen-2: Probably damaging SIFT: Deleterious SDM: Stabilizing *NLRP5* rs36118060 NM_153447.4: c.3584G > A; p.Arg1195Gln AF: 0.145 GF: 0.022 PolyPhen-2: Benign SIFT: Tolerated SDM: Destabilizing	M: het P: wt F: wt M: hom P: het F: wt M: hom P: het F: wt	Hypomethylated loci: *KCNQ1OT1, PLAGL1, IGF1R, GNAS-AS1* Hypermethylated loci: none	Proband born by in vitro fertilization	None	Macroglossia, mild asymmetry, macrosomia, neonatal hypoglycaemia, atopic eczema, gestational diabetes BWS score: 6
5	Female, 22	*NLRP2* rs61735086 NM_017852.4: c.1681G > A; p.Ala561Thr AF: 0.000 GF: 0 PolyPhen-2: Benign SIFT: Tolerated SDM: Destabilizing *NLRP2* rs17699678 NM_017852.4: c.662C > T; p.Thr221Met AF: 0.110 GF: 0.012 PolyPhen-2: Possibly damaging SIFT: Deleterious SDM: Stabilizing	M: het P: wt F: wt M: het P: het F: wt	Hypomethylated loci: *KCNQ1OT1,* *NAP1L5, NNAT,* *GNAS-XLas, GNAS A/B, GNAS-AS* Hypermethylated loci: *ZNF331-DMR2, GNAS-NESP*	One miscarriage	Two daughters	Macroglossia, hyperinsulinism, hypoglycaemia, hepato/splenomegaly, nevus flammeus (head, neck), maxillary hypoplasia, neonatal anaemia, respiratory distress, round face. BWS score: 6
6	Female, 14	*NLRP2* rs17699678 NM_017852.4: c.662C > T; p.Thr221Met AF: 0.110 GF: 0.012 PolyPhen-2: Possibly damaging SIFT: Deleterious SDM: Stabilizing	M: het P: het F: wt	Hypomethylated loci: *KCNQ1OT1, NAP1L5, PLAGL1, PEG10, MEST, GNAS-XLas, GNAS A/B, GNAS-AS1* Hypermethylated loci: *GNAS-NESP*	None	None	Macroglossia, exomphalos, perinatal hypoglycaemia, macrosomia, anterior creases in the ear, nevus flammeus (neck), round face, haemangioma. BWS score: 7
7	Male, 15	*PADI6* rs74834315 NM_207421.4: c.775G > A; p.Val259Ile AF: 0.001 GF: 0 PolyPhen-2: Benign SIFT: Deleterious SDM: Destabilizing	M: het P: het	Hypomethylated loci: *KCNQ1OT1,* *IGF1R, SNU13* Hypermethylated loci: none	None	None	Macroglossia, macrosomia, round and coarse face with prominent forehead, antimongoloid palpebral fissures and later on advanced bone age. BWS score: 3
8	Female, 4	*NLRP2* rs147585490 NM_017852.4: c.1055 T > G; p.Ile352Ser AF: 0.002 GF: 0 PolyPhen-2: Possibly damaging SIFT: Deleterious SDM: Destabilizing	M: het P: wt	Hypomethylated loci: *GNAS-XLas, GNAS A/B, GNAS-AS,* *DIRAS3:Ex2, PLAGL1, MEST, ERLIN2, PEG13, IGF1R, SNU13* Hypermethylated loci: *GNAS-NESP, ZDBF2/GPR1*	Proband born by in vitro fertilization	None	Early onset obesity, intellectual disability
9	Female, 35	*NLRP2* rs61735077 NM_017852.4: c.1060A > G; p.Ile354Val AF: 0.008 GF: 0.000 PolyPhen-2: Benign SIFT: Tolerated SDM: Destabilizing	M: het P: wt	Hypomethylated loci: *GNAS-XLas, GNAS A/B, GNAS-AS, DIRAS3, FAM50B, MEST, MCTS2P* Hypermethylated loci: *GNAS-NESP, NDN*, *ZNF597*	None	None	Truncal obesity, hypothyroidism, hypercholesterolemia, occasional high PTH levels
10	Male, 8	*NLRP2* rs4306647 NM_017852.4: c.1091G > A; p.Arg364Lys AF:0.041 GF: 0.001 PolyPhen-2: Possibly damaging SIFT: Tolerated SDM: Destabilizing	M: het P: wt	Hypomethylated loci: *GNAS-XLas, GNAS A/B, GNAS-AS, PEG10, PEG13, ZNF331* Hypermethylated loci: *GNAS-NESP*	One miscarriage	None	Short stature, mild global developmental delay, PTH resistance with hypocalcaemia and hyperphosphataemia

In third column AF: Allele frequency in European population; GF: frequency of the homozygous genotype of the variant in European population. In fourth column: M = mother, P = Proband, S = Sister, F = Father, na: not available

MLID was identified in peripheral blood leukocyte (PBL) DNA by employing the multi-locus methylation-specific multiple ligation-dependent probe amplification (MS-MLPA) assay ME034-B1 (MRC-Holland, Amsterdam, The Netherlands) or the Illumina Infinium methylation (HM450k, EPIC or custom) bead chip array. Altered methylation levels of at least one germline DMR in addition to that diagnostic for BWS or PHP1B (*KCNQ1OT1*:TSS-DMR and *GNAS A/B*:TSS-DMR, respectively) were found in the probands of ten families (Additional file 3: Table S1). The list of DMRs affected by methylation disturbances in each proband is reported in Table 1 and the methylation values in Additional file 3: Table S1).

The methylation defect was partial in most of the DMRs and the number of hypomethylated DMRs was more prevalent compared to the hypermethylated DMRs. The number and methylation levels of the affected DMRs were variable even between the probands sharing maternal effect variants in the same gene. In addition to the diagnostic locus, the most frequently hypomethylated loci were *GNAS* (5/7) and *PLAGL1* (5/7) in the patients affected by BWS, *DIRAS* (2/3), *MEST* (2/3) and *PEG13* (2/3) in the patients affected by PHP1B. In two PHP1B patients, the proband of family 9 and the proband of family 10, the *GNAS* DMRs were more severely hypomethylated than in the BWS patients. In addition, the *KCNQ1OT1*-TSS:DMR was found hypomethylated in BWS but not in PHP1B, while *PEG13* was hypomethylated only in PHP1B. Other imprinted loci were hypomethylated in less than half of the patients.

Overall, the methylation data demonstrated a profile of apparently randomly disturbed methylation of the imprinted DMRs in all the probands rather than a specific epi-signature for each single SCMC gene.

### Identification of the maternal variants of the SCMC genes

Since maternal-effect variants of the SCMC genes have been previously associated with MLID, we looked for variants of these genes in the mothers of the identified probands, by either whole-exome sequencing (WES) or SCMC gene targeted Sanger sequencing analyses (see also Methods section). We selected all rare (allele frequency < 1%) and common (allele frequency > 1%) variants that were predicted to be damaging/deleterious by the bioinformatic prediction tools PolyPhen-2 [34, 35] and SIFT [36] or destabilizing by SDM [37]. Overall, we identified thirteen putatively deleterious maternal missense variants in ten pedigrees. According to the criteria of the American College of Medical Genetics and Genomics (ACMG) [38] ten of these variants were classified as variants of uncertain significance (VUS), and three as benign because their frequency was higher than 5% in the general population. Possible limitations of the ACMG criteria for interpreting pathogenicity of maternal-effect variants are described in the Discussion.

The genetic, epigenetic and phenotypic features of the families are summarized in Table 1, while all the characteristics of the variants including ACMG classification are reported in Additional file 4: Table S2. The variants occurring in each family are described in more detail below.

*Family 1*. A novel variant of *KHDC3L* was found in homozygosity in the proband 1’s mother (Additional file 2: Fig. S1a). This is a missense variant (chr 6, c.296 C > G; p.Thr99Arg) located within exon two and affecting the threonine in position 99 of the protein. This residue is not evolutionarily conserved, but in silico analyses indicated its harmful effect. We observed that this position is usually held by amino acids containing a methyl group, most frequently valine and threonine, two isosteric residues (Additional file 2: Fig. S1b, top). According to the AlphaFold [39] Protein Structure Database [40] model of KHDC3L, Thr99 belongs to an alpha helix and entertains a hydrophobic interaction (via its methyl group) with residue Ile103 and a hydrogen bond (via its hydroxyl group) with residue Ser70 (Fig. S1b, bottom). Both residues, as threonine, are needed to maintain multiple intramolecular interactions with other residues. The substitution of Thr99 with a charged amino acid, in our case arginine, likely disrupts these interactions, destabilizing the protein structure with harmful effects, as predicted by PolyPhen-2, SIFT and SDM tools (Additional file 4: Table S2).

The *KHDC3L* c.296C > G; p.Thr99Arg variant was present in heterozygosity in the proband and maternal grandmother and absent in the father and maternal grandfather (Table 1 and Additional file 2: Fig. S1a). To explain the homozygosity of this variant in the mother, a SNP-array analysis was performed to detect the presence of uniparental disomy or deletions on chromosome 6q. The results demonstrated a normal pattern on most chromosomes, but revealed the presence of long regions of heterozygosity mixed to long regions of loss of heterozygosity along the entire chromosome 6 (Additional file 2: Fig. S1c). This profile corresponds to a mixed heterodisomic/isodisomic UPD that is likely caused by physiological meiotic recombination followed by non-disjunction and disomic gamete formation, and zygotic trisomy rescue after fertilization [41]. The *KHDC3L* locus was located in one of the three isodisomic intervals, thus explaining the homozygous genotype of the mother. To determine the parental origin of the UPD, we analysed the DNA methylation of the imprinted *PLAGL1* DMR that is also located on chr 6. Because this DMR was totally methylated while the DMRs located on different chromosomes showed a normal 50% methylation level, we concluded that the upd(6) was of maternal origin (Additional file 2: Fig. S1d). Analysis of the grandparental DNAs by SNP-array confirmed maternal upd of the entire chromosome 6 in the proband’s mother of family 1 (Additional file 5: Table S3).

Concerning the phenotype, no particular clinical sign was observed in the mother. She was born at term from uneventful pregnancy, her birth length and weight were referred to be normal and her final adult height was 158 cm. She had normal cognitive and physical development and she has not reported any significant diseases so far.

**Fig. 1:**
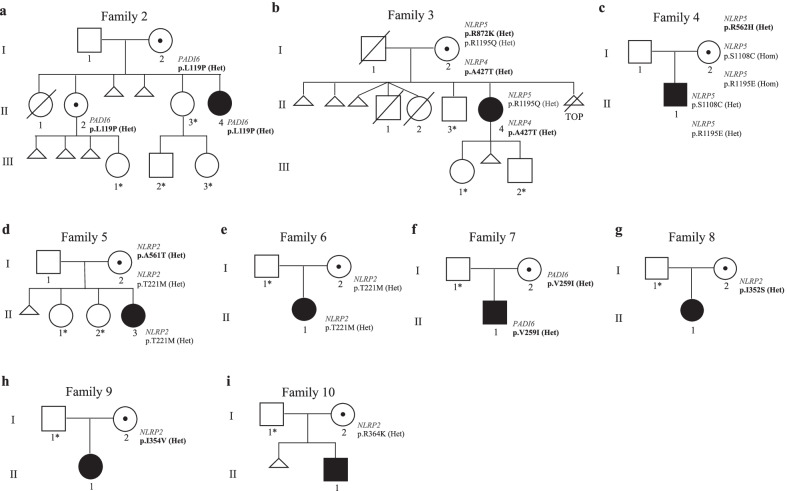
Pedigrees of families 2–10. Black filled symbol represents the probands affected by BWS or PHP1B, black central dots the unaffected carrier mothers. Triangles: spontaneous miscarriage. Triangle with line: voluntary termination of pregnancy. The variants in bold are rare (AF < 0.01), those in regular font style are common (AF > 0.01). The asterisks indicate the family members whose DNA was not available for genetic analyses

*Family 2.* A novel variant was identified in *PADI6* and found in heterozygosity in the proband 2’s mother. This is a missense mutation (c.356C > T; p.Leu119Pro) in exon 3, affecting the leucine in position 119 of the protein close to the end of the N-terminal domain, and predicted to be strongly destabilizing according to the score of SDM prediction (Additional file 4: Table S2). The variant was inherited by the proband and her healthy second sister (II-2, Fig. 1a) who had a healthy daughter after three consecutive miscarriages (Table 1 and Additional file 1: supplemental information). Methylation was tested in both the proband’s sister II-2 (by multi-locus MS-MLPA and methylome EPIC 850 k array) and mother (by multi-locus MS-MLPA) was found to be normal (data not shown).

*Families 3–7.* Rare missense variants affecting several genes of the SCMC and belonging to the NLRP family were found in heterozygosity in the probands’ mothers of families 3–6 (Fig. 1b–e). In particular, p.Arg872Lys of *NLRP5* was found in family 3, p.Arg562His of *NLRP5* in family 4 and p.Ala561Thr of *NLRP2* in family 5 (Table 1). These three women were also carriers of common variants affecting the same genes. P.Arg1195Gln of *NLRP5* was in heterozygosity in the proband’s mother of family 3, p.Ser1108Cys and p.Arg1195Gln of *NLRP5* were in homozygosity in the proband’s mother of family 4 and p.Thr221Met of *NLRP2* was in heterozygous state in the proband’s mother of family 5. Segregation analysis in the trios (Table 1 and Fig. 1) demonstrated that the rare and the common variants were present in compound heterozygosity in the mother of family 5 (Fig. 1d) and possibly in the mother of family 3 (Fig. 1b). Indeed, only the common variants were inherited by the probands and no variant was present in the father of family 5. However, in family 3 because of missing information on the father’s genotype, the possibility that the common variant was transmitted by the father to the proband cannot be ruled out. Furthermore, a rare variant of *NLRP4* (p.Ala427Thr) was found in heterozygosity in both the mother and proband of family 3. The mothers of pedigrees 6–7 were heterozygous for single variants: p.Thr221Met of *NLRP2* in family 6 (Fig. 1e) and p.Val259Ile of *PADI6* in family 7 (Fig. 1f).

*Families 8–10.* Missense variants of *NLRP2* were identified in heterozygosity in the mothers of the three patients affected by PHP1B: p.Ile352Ser in family 8, p.Ile354Val in family 9 and p.Arg364Lys in family 10 (Fig. 1g–i).

Overall, the sequencing data demonstrated that the ten mothers were carriers of at least one putatively damaging variant affecting the SCMC genes. In some of them, a rare variant was in homozygosity or in compound heterozygosity with another rare or common putatively damaging variant, or variants affecting more than one SCMC gene were present in the same woman.

## Discussion

In this study, we report ten new cases of MLID and clinical features of either BWS or PHP1B, in which segregate putatively damaging maternal-effect missense variants of the SCMC genes, further extending the list of genes and IDs associated with maternal variants. These variants were classified as VUS or benign according to the ACMG criteria, but their pathogenic damaging effect could have been underestimated for the following reasons: i) maternal-effect variants are present in the maternal genome but affect the offspring phenotype; ii) no effect on male reproduction has been reported so far; iii) they are associated with variable expressivity and incomplete penetrance of the molecular (methylation disturbance) and clinical phenotype of the offspring of carrier mothers; iv) all the previous points might explain why some SCMC variants, although potentially damaging, have high frequency in the general population.

*The phenotypes*. SCMC variants have been associated with phenotypes of different severity ranging from female infertility, RHM and recurrent miscarriages, to IDs with MLID in the offspring. It has been proposed that the severity of the reproductive outcome is dependent on the impact of the variants on protein function, so that the variants completely inactivating the gene product cause non-viable phenotypes, while the hypomorphic variants result in MLID [17, 18]. The results obtained from the BWS family 1 strongly support this hypothesis. In this case, the proband’s mother carries a hypomorphic variant of *KHDC3L* in homozygosity, while biallelic severely inactivating pathogenic variants of this gene have been associated with RHM and recurrent pregnancy loss so far [28, 29, 42–45].

Some of the pedigrees reported in this study and segregating maternal-effect SCMC missense variants include recurrent miscarriages in addition to ID phenotypes in the offspring. Intrafamilial heterogeneous reproductive outcomes have been reported before and could be a consequence of the multiple cellular functions of the SCMC components [20, 27]. However, as no DNA from the miscarriages were available for genetic analysis, we cannot rule out causes other than the SCMC gene variants for the pregnancy losses.

Interestingly, two patients affected by PHP1B showed also intellectual disability (proband of family 8) or mild global developmental delay (proband of family 10), two clinical features already reported associated with several cases of MLID [13, 14, 16–18].

In all our families, the viable progeny shows a variable number of hypomethylated imprinted germline DMRs (Additional file 3: Table S1). Since the methylation changes are often partial and likely in the mosaic form, it is possible that the clinical presentation of the affected individuals depends on which imprinted locus is more strongly affected (epidominance hypothesis) [7, 17]. Interestingly, this methylation variability appears to be more characteristic of the SCMC-MLID cases than the *ZFP57*-TNDM-MLID cases in which some DMRs are more consistently affected. This could be caused by impairment of the different functions of these trans-acting factors: weaker binding of zygotic variants of ZFP57 to the methylated allele at the imprinted DMRs or a general methylation establishment/maintenance defect caused by maternal SCMC variants.

Hypermethylation is also observed, mostly affecting paternally methylated secondary DMRs if their respective maternally methylated germ-line DMRs are hypomethylated (e.g. *GNAS-NESP*, *ZNF597*, and *ZDBF2/GPR1*). Mild hypermethylation affects also some maternal germ-line DMRs, likely as consequence of a defective general mechanism of DNA methylation maintenance in early embryogenesis or a secondary effect of the hypomethylation of other loci.

*The genotype*. If the SCMC variants act as dominant or recessive mutation is debated. While the variants of *NLRP7*, *KHDC3L* and *PADI6* that were associated with RHM have consistently been found in homozygosity or compound heterozygosity, more than half of the SCMC pathogenic variants reported in MLID cases have been found in heterozygosity in the probands’ mothers [16–18]. In the present study, putatively damaging maternal SCMC gene variants were found in either homozygosity or simple or compound heterozygosity. In particular, rare heterozygous variants were found in families 2 and 6–10, a rare variant was present in homozygosity in family 1 and rare variants were in compound heterozygosity with common putatively damaging variants in families 3–5. This suggests that common variants may play a role in the aetiology of the MLID if potentially deleterious and present in combination with rarer variants. Alternatively, heterozygous variants might act as dominant-negative mutants [17] or by exerting an additive effect in combination with variants affecting further SCMC components or other unidentified genes. In this regard, we found heterozygous maternal variants of more than one SCMC gene in family 3. It is also worth mentioning that ours is the first report of SCMC variants associated with PHP1B.

*KHDC3L*. The proband of family 1 represents the first case of MLID associated with a maternal variant of *KHDC3L*. This finding raises a question concerning the role of this protein in imprint establishment or maintenance. The involvement of *KHDC3L* in RHM, a gestational abnormality with broad loss of maternally but not paternally methylated imprints, implicates *KHDC3L* in oocyte-specific methylation establishment. Also, the methylation abnormalities of the family 1 proband appear to be restricted to the maternal germ-line DMRs suggesting an oocyte origin of the defect. However, because some DMRs are only partially hypomethylated in this patient, a role of *KHDC3L* in post-zygotic imprint maintenance cannot be excluded. Consistent with this hypothesis, also for *NLRP7* a role in both imprint establishment and maintenance has been proposed, since maternal-effect variants of this gene were found in both RHM and MLID cases with hypomethylated paternal DMRs [17, 32].

Interestingly, in family 1 two rare events, homozygosity for the rare putatively damaging *KHDC3L* variant and upd(6)mat, have occurred in the same individual, the proband’s mother. Although UPD is a possible mechanism underlying recessive phenotypes of rare pathogenic variants, the hypothesis that these two rare events are causally linked is plausible. Constitutional UPD of an entire chromosome is usually caused by trisomic rescue of a zygote derived from fertilization of a disomic gamete [41, 46]. In this family, the hypermethylation of *PLAGL1*, the segregation of the *KHDC3L* variant and the SNP-array data indicate that the disomic gamete has derived from the maternal grandmother. A number of evidences support the hypothesis that the SCMC members, and particularly *KHDC3L*, are involved in aneuploidies [27, 45, 47, 48]. Thus, it is possible that the *KHDC3L* variant is responsible for the occurrence of gametic non-disjunction and disomy in the maternal grandmother and upd(6) in the mother. Consistent with the finding that upd(6)mat is not necessarily associated with pathological conditions [47, 49] (http://upd-tl.com/upd.html), no significant clinical feature was reported for this woman.

*PADI6.* Similar to a maternal-effect variant reported by Begemann et al. [17], the p.Val259Ile variant found in family 7 affects the second domain (the PAD middle domain) of the PADI6 protein. In contrast, all the other MLID-associated pathogenic variants reported so far fall within the third domain of this protein (the Protein Arginase Deiminase domain) [18]. Differently, the novel missense Leu119Pro variant found in family 2 affects the first domain (the PAD N-terminal) suggesting that also this part of the protein might have a role in the establishment/maintenance of the methylation at the imprinted loci. The p.Leu119Pro was predicted to strongly destabilize the protein and this might explain the recurrent pregnancy loss of the family 2 (Fig. 1a). However, despite the inheritance of the variant and the recurrent pregnancy loss, no methylation defect of the imprinted DMRs was found in the proband’s sister II-2. This is consistent with the incomplete penetrance of the epigenotype and phenotype that often characterizes the SCMC-MLID pedigrees [17, 18], and that might be explained by a polygenic hypothesis, according to which, further unknown maternal or zygotic factors may be involved in MLID aetiology, by acting as modifiers of the phenotype of the maternal SCMC genes.

*NLRPs*. Although several variants of *NLRP2* and *NLRP5* have been found in MLID pedigrees, none of the variants described in the present study have been previously reported associated with IDs or reproductive conditions. Interestingly, the variant p.Thr221Met found in pedigrees 5–6 was present at higher frequency than that reported in dbSNP in a group of 94 women with diagnosis of unexplained infertility (Allele frequency: 0.313 vs 0.061) [50].

The variant of *NLRP4* found in the proband’s mother of family 3 represents the first maternal-effect variant of this gene found associated with MLID. This finding supports a role of NLRP4 as a member of the human SCMC. *NLRP4* is orthologous to the mouse *Nlrp4f* that encodes a protein with the same cellular distribution of Nlrp5 and is required for cytoplasmic lattices formation and organelle distribution in oocytes [23, 26]. Notably, this mother had serious reproductive problems (Fig. 1b and Additional file 1: Supplemental information), and she was the only one carrying putatively damaging variants affecting more than one SCMC gene (*NLRP4* and *NLRP5*). This suggests an additive effect of the variants on the impairment of the SCMC function resulting in a more severe reproductive outcome.

Further studies on larger cohort of MLID families and animal models are needed to clarify the role of this complex in human reproduction and the impact that the rare and less rare missense variants of the SCMC genes have on methylation disturbance and imprinting disorders.

## Conclusions

In summary, we identified ten new cases of MLID, seven clinically affected by BWSp and three by PHP1B. By WES and SCMC gene targeted sequencing, we identified in their mothers thirteen variants of genes encoding components of the SCMC, including NLRP2, NLRP4, NLRP5, PADI6 and KHDC3L. Among the variants, two were novel, seven rare and four common, but all were predicted to be harmful. Further, by methylome array we showed methylation profiles of the MLID patients characterized by variable number and methylation level of affected DMRs, demonstrating lack of a specific epi-signature for each single SCMC gene. Overall, these data increase our knowledge on the link between the maternal-effect variants of the SCMC genes and the MLID-associated IDs. All this information should be considered for genetic counselling of MLID families to improve molecular diagnosis and prediction of the recurrence risk of the IDs.

## Methods

### Analyses of epigenetic and genetic variants

Genomic DNA was isolated from PBL by the salting-out procedure [51].

*MS-MLPA* analysis was performed by SALSA MS-MLPA Kit (MRC-Holland, Amsterdam, The Netherlands) according to the manufacturer’s protocol: Probemix ME034 for multi-locus imprinting was applied to BWS cases (probands 1–7) [49]. Probemix ME031for GNAS locus was applied to PHP1Bcases (probands 8–10).

*SNP-array* on DNA mother and maternal grandparents of proband 1 was performed as previously reported [49].

*DNA Sequencing*. Whole-exome sequencing was performed on DNA of mothers of families 1–6 at IGATech Service (Udine, Italy). Nextera Flex for Enrichment solution (Illumina, San Diego, CA) in combination with “SureSelect Human All Exon V7” probes (Agilent, Santa Clara, CA) was used for library preparation and exome enrichment, targeting 50 Mb of human exonic content. The samples were quantified and quality tested using the Qubit 2.0 Fluorometer (Invitrogen, Carlsbad, CA) and Agilent 2100 Bioanalyzer (Agilent Technologies, Santa Clara, CA). Libraries were sequenced on NovaSeq 6000 (Illumina, San Diego, CA) in 150 pair-end mode. The bioinformatic analysis was performed as previously reported [18].

Exon-PCR and Sanger sequencing of all known SCMC genes (TLE6, NLRP2, NLRP7, NLRP5, OOEP, PADI6 and KHDC3L) was performed on PBL DNA from both mothers and probands of families 7–10.

Segregation analysis of variants in the family members was performed by Sanger sequencing. In silico prediction of variant pathogenicity by bioinformatic tools was performed as previously reported [18]. The variants were also classified following the ACMG standards and guidelines [38].

*Methylome array* was performed using Illumina Infinium Methylation BeadChip kit on PBL DNA of all patients. Probands 1–6 were analysed by the Epic 850 k kit at BIODIVERSA srl Service (Milan, Italy) as previously reported [18]. Briefly, DNA of probands and twelve non-affected individuals (7 females and 5 males, aged between 2 and 32) used as was subjected to bisulfite conversion and methylation array processing by the EPIC BeadChip array, following the manufacturer’s protocol. Array data were analysed in Rstudio (v4.1.0) using the Bioconductor package “ChAMP” (v.2.22.0). After importing the “idat” files in RStudio and filtering them using “champ.load”, we obtained the Beta value matrix, which retains 713,288 probes. We then applied “BMIQ” normalization and batch correction using “champ.norm” and “champ.runCombat” functions, respectively.

The methylomes for patients 7 and 8 were generated using the Illumina Infinium Human Methylation 450 and EPIC 850 BeadChip arrays, respectively, and compared to 20 control PBL samples. Bisulphite conversion was performed according to the manufacturer’s recommendations for the Illumina Infinium Assay (EZ DNA methylation kit, ZYMO, Orange, CA) and hybridization following the Illumina Infinium HD methylation protocol at genomic facilities of the Cancer Epigenetics and Biology Program (Barcelona, Spain). Before analysing the data, we excluded possible sources of technical biases that could influence results. We applied signal background subtraction, and interpolate variation was normalized using default control probes in BeadStudio (version 2011.1_Infinium HD). We discarded probes with a detection p value > 0.01, containing single nucleotide polymorphisms (SNPs) within the interrogation or extension base, those mapping to sex chromosomes as well as those with potential cross-reaction due to multiple sequence homologies, which resulted in 365,359 retained probes, 658 mapping to imprinted DMRs common to both platforms. In-house bioinformatics R scripts were utilized for statistical comparisons.

Probands 9 and 10 were analysed with a custom Illumina Veracode GoldenGate methylation array and are already described in Court et al. [7] as PHP0081 and PHP0089, respectively. Twenty-eight DNA samples from phenotypically normal individuals aged between 1 and 35 years (17 females, 11 males) were used as control for sample comparison using the custom array platform, with methylation values of eight different complete chromosome UPDs (for chr:6, 7, 14, 15 and 20) and uniparental diploidy samples employed to define extreme methylation profiles [7].

To investigate the methylation profile at the imprinted DMRs, we downloaded the coordinates from http://www.humanimprints.net/, identifying every probe across the imprinted regions. After filtering out all the iDMRs with low coverage (< 4 probes), we calculated the average methylation level for each region, considering as statistically significant those exceeding three standard deviations from the average of the controls.

### Protein bioinformatics

To study Thr99 conservation, we used the NCBI blast service on the Swissprot database with the KHDC3L UniProt accession id (Q587J8) as the input sequence identifier. We aligned the blast output with clustalW to produce a Multiple Sequence Alignment (MSA) and then visualized the MSA using WebLogo. The effect of the deleterious variants was predicted using the sequence-based tools SIFT [34] and PolyPhen-2 [35, 36] and the SDM tool for the structure-based analysis of substitution tolerance [37]. Chimera version 1.15 (build 42,258) was used to visualize the KHDC3L PDB from the AlphaFold Protein Structure Database [39, 40].

## Supplementary Information

**Additional file 1:** Supplemental information. Clinical features of the patients described in the present study.

**Additional file 2: Figure S1.** Genetic analyses of family 1. (a) Pedigree. (b) top: WebLogo of KHDC3L orthologues MSA in the 70-103 position range. Y-axis: the probability of finding a specific residue in each position; dashed lines mark relevant model-based interaction between residuals (black: T99-I103 hydrophobic, blue: T99 - S70 hydrogen bond); bottom: AlphaFold Protein Structure Database model of KHDC3L showing T99 and the interactions: hydrophobic T99 - I103 via methyl group (black) and hydrogen bond T99 - S70 via hydroxyl (blue). (c) SNP-array results of chromosome 6. (d) MS-MLPA of the proband’s mother (II-2). The red arrow indicates the histogram of *PLAGL1* DMR showing 100% of methylation.

**Additional file 3: Table S1.** DNA methylation values of imprinted loci obtained by methylome array.

**Additional file 4: Table S2.** List of the SCMC variants identified in the probands mothers.

**Additional file 5: Table S3.** List of the SNP genotypes of the whole chromosome 6 obtained by SNP- array analysis on mother and maternal grandparents of proband 1.

## Data Availability

Methylation array data that support the findings of this study have been deposited under accession code GSE195873 and G19825 in the Gene Expression Omnibus repository.

## Abbreviations

BWS: Beckwith–Wiedemann syndrome
DMRs: Differentially methylated regions
ICs: Imprinting centres
IDs: Imprinting disorders
MLID: Multi-locus imprinting disturbances
PBL: Peripheral blood leukocytes
PHP: Pseudohypoparathyroidism
TNDM: Transient neonatal diabetes mellitus
SCMC: Sub-cortical maternal complex
UPD: Uniparental disomy
VUS: Variant of unknown significance
WES: Whole-exome sequencing
