# Supplementary material for: Novel genetic variants of KHDC3L and other members of the subcortical maternal complex associated with Beckwith–Wiedemann syndrome or Pseudohypoparathyroidism 1B and multi-locus imprinting disturbances
Source: Clin Epigenetics. 2022 May 28;14:71. doi: 10.1186/s13148-022-01292-w (PMC9148495; doi:10.1186/s13148-022-01292-w)
Supplement: Supplementary file 1 — Additional file 1: Supplemental information. Clinical features of the patients described in the present study. [file 13148_2022_1292_MOESM1_ESM.pdf]

Supplemental information on the families described in this study

The proband of family 1 (III-1, Fig. S1) was a 24-year-old woman whose clinical history and molecular diagnosis was previously reported (Bliek et al., 2009). At the last follow-up a recently arisen left pantonal transmissive hearing loss and otosclerosis were reported. Hepato- and splenomegaly at a border-line level were also reported.

The proband of family 2 (II-4, Fig. 1a) was a 39-year-old woman, the youngest of an offspring composed of four daughters and two miscarriages occurred between the second and the third birth. At a recent follow-up a bilateral hearing loss was reported. Her eldest sister (II-1, Fig. 1a) died when she was 5 years old because of peritonitis. The second sister (II-2, Fig. 1a), a 47-year-old woman who joined this study, had three consecutive miscarriages and a healthy baby girl (III-1, Fig. 1a) in her fourth pregnancy. The third sister, whose DNA was not available for genetic analyses, had two healthy children, a boy and a girl.

The proband of family 3 (II-2, Fig. 1b) was a 40-year-old woman, mother of two healthy children, a 10-year-old girl and a 3-year-old boy. She had also a miscarriage between the first and second birth. Because of infertility problems her mother had the first four pregnancies by ovarian stimulation at the age of 20-21year old. The first two were spontaneously lost during the first trimester, the third was a triplet: one miscarriage at the third month of gestation, two children born at 6 months of gestation and died few hours/days later. The fourth pregnancy was full term birth of a healthy son. The lady had two more spontaneous pregnancies without ovarian stimulation, the first was full term birth of the proband, the second was voluntarily terminated (Fig. 1b).

The proband of family 4 (OGS128) was a 15-year-old boy born by in vitro fertilization.

The proband of family 5 (OGS653) was a 22-years old woman, the youngest of an offspring composed of three daughters and one miscarriage occurred in the first pregnancy of the couple. The index case was born by natural conception and at term induced due to reduce fetal movements. Anthropometrical parameters showed increased weigh, length above +2 standard deviation. She suffered with neonatal hypoglycemia with hyperinsulinism and anemia, and physical examination showed nevus flammeus, macroglosia, maxilla hypoplasia with relative prognathism, interatrial communication (IAC) and transitory respiratory distress. Additional clinical features included round face, diastema of teeths, and hepatomegaly with splenomegaly.

The proband of family 6 (OGS1052**)** was a 14-years old female, she has one older normal sibling. Delivery was at term by cesarean due to fetal weight (4,900kg at birth). At perinatal stage, she presented with generalized overgrowth, round face, macroglossia, anterior creases in the ear, hemangioma, omphalocele, nevus flammeus at neck and perinatal hypoglycemia.

The proband of family 7 (OGS1344) was a 15-years old male, he has an older sibling. Delivery was at term by natural conception, and antropometric parameters at birth were: weight: 3,470gr, length: 49cm and HC: 34,5cm. At perinatal stage he presented with generalized overgrowth, round and coarse face with prominent forehead, macroglossia antimongoloid palpebral fissures and lateron; advanced bone age was also detected.

The index of family 8 (PHP1138), a $4^{5/12}$ -years-old girl, was referred to discard PHP1A because of early-onset obesity and mental retardation even if her phosphocalcium metabolism was normal. When testing for *GNAS* deletions, incidentally methylation alteration was detected. The methylation study was extended to analyze further DMRs. She was conceived by ART.

The index of family 9 (PHP0081) was a 35-year-old woman who was followed since she was 19 with clinical suspicion of Cushing syndrome due to her phenotype. Normal cortisol values, both basal and after stimulation discarded the suspicion. When she was 30, hypothyroidism was detected. The presence of obesity and hypothyroidism pointed to PHP1B and calcium/phosphorous metabolism analyzed several times with occasional high PTH levels accompanied by calcium at the lower normal limit. She also presented hypercholesterolemia.

The proband of family 10 (PHP0089) is a $7^{10/12}$ -years-old boy recruited because of short stature. His mother presented hypothyroidism treated with hormone replacement. She suffered a previous miscarriage and in the gestation that gave birth to the proband she also presented a miscarriage alarm at 12 weeks of gestation. The proband presented mild global developmental delay. Complete hormonal testing revealed high PTH levels associated with hypocalcaemia and hyperphosphataemia and clinical diagnosis of PHP1B was established.
