# Supplementary material for: Novel genetic variants of KHDC3L and other members of the subcortical maternal complex associated with Beckwith–Wiedemann syndrome or Pseudohypoparathyroidism 1B and multi-locus imprinting disturbances
Source: Clin Epigenetics. 2022 May 28;14:71. doi: 10.1186/s13148-022-01292-w (PMC9148495; doi:10.1186/s13148-022-01292-w)
Supplement: Supplementary file 2 — Additional file 2: Figure S1. Genetic analyses of family 1. (a) Pedigree. (b) top: WebLogo of KHDC3L orthologues MSA in the 70-103 position range. Y-axis: the probability of finding a specific residue in each position; dashed lines mark relevant model-based interaction between residuals (black: T99-I103 hydrophobic, blue: T99 - S70 hydrogen bond); bottom: AlphaFold Protein Structure Database model of KHDC3L showing T99 and the interactions: hydrophobic T99 - I103 via methyl group (black) and hydrogen bond T99 - S70 via hydroxyl (blue). (c) SNP-array results of chromosome 6. (d) MS-MLPA of the proband’s mother (II-2). The red arrow indicates the histogram of PLAGL1 DMR showing 100% of methylation. [file 13148_2022_1292_MOESM2_ESM.pdf]

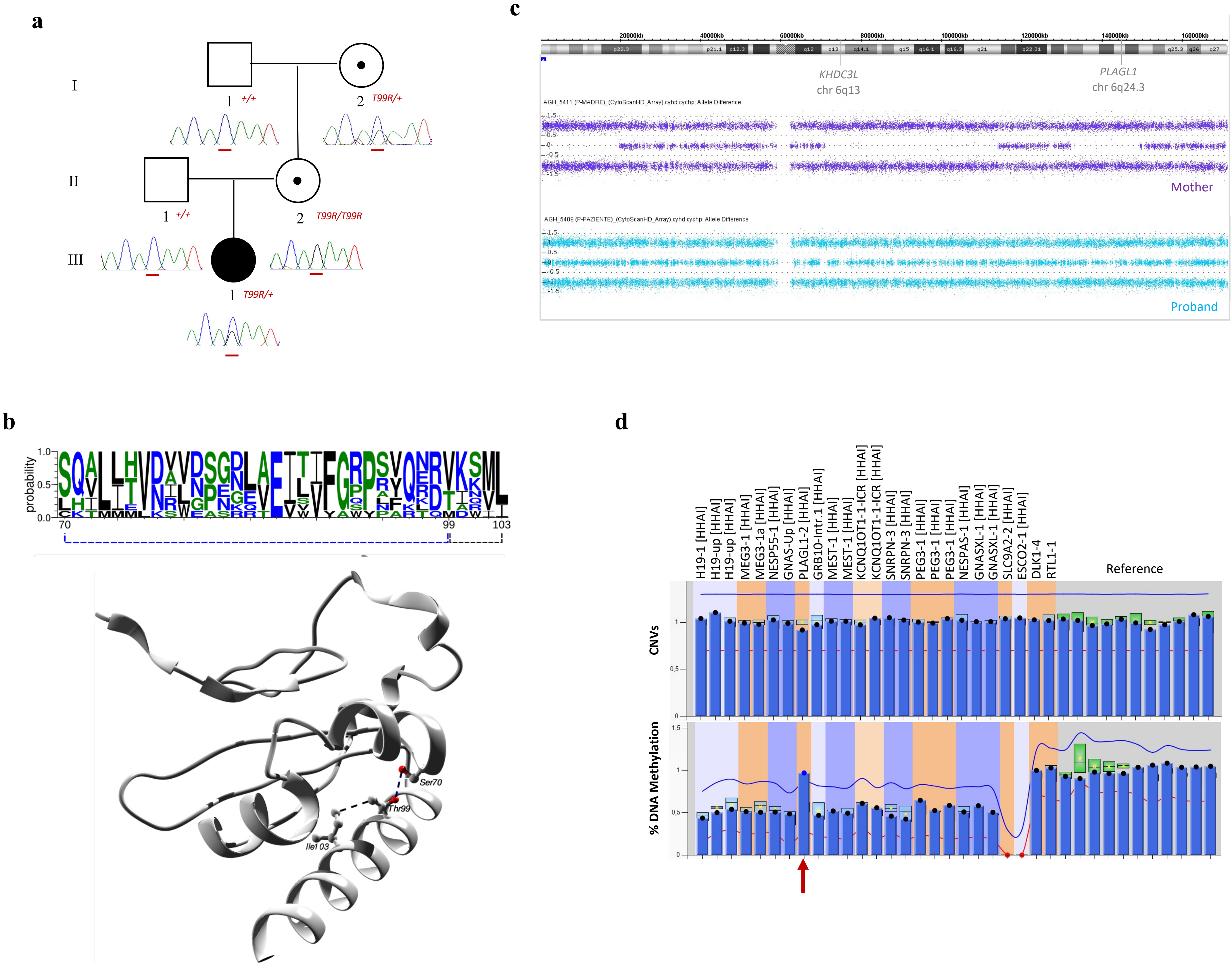

Figure S1. Genetic analyses of family 1. (a) Pedigree. (b) top: WebLogo of KHDC3L orthologues MSA in the 70-103 position range. Y-axis: the probability of finding a specific residue in each position; dashed lines mark relevant model-based interaction between residuals (black: T99-I103 hydrophobic, blue: T99 - S70 hydrogen bond); bottom: AlphaFold Protein Structure Database model of KHDC3L showing T99 and the interactions: hydrophobic T99 - I103 via methyl group (black) and hydrogen bond T99 - S70 via hydroxyl (blue). (c) SNP-array results of chromosome 6. (d) MS-MLPA of the proband's mother (II-2). The red arrow indicates the histogram of *PLAGL1* DMR showing 100% of methylation.
