# Supplementary material for: Novel genetic variants of KHDC3L and other members of the subcortical maternal complex associated with Beckwith–Wiedemann syndrome or Pseudohypoparathyroidism 1B and multi-locus imprinting disturbances
Source: Clin Epigenetics. 2022 May 28;14:71. doi: 10.1186/s13148-022-01292-w (PMC9148495; doi:10.1186/s13148-022-01292-w)
Supplement: Supplementary file 4 — Additional file 4: Table S2. List of the SCMC variants identified in the probands mothers. [file 13148_2022_1292_MOESM4_ESM.pdf]

Table S2. List of the SCMC missense variants and prediction of their effect on the protein structure and function

Gene	Position (hg19)	Alleles	Variant ID (dbSNP)	[a]Allele frequency (ALL)	[b]Allele frequency (NFE)	[c]Genotype frequency (NFE)	Conservation in orthologous proteins	AA	[d]ACMG classification	Prediction		
										PolyPhen-2	SIFT	SDM (DDG kcal/mol)
KHDC3L	Chr 6:74072944	C>G	novel	-	-	-	No	T99R	Uncertain significance	Possibly damaging (0.908)	Deleterious (0)	Destabilizing (-0.51)
PADI6	Chr 1:17701983	T>C	novel	-	-	-	Yes	L119P	Uncertain significance	Probably damaging (1)	Deleterious (0)	Destabilizing (-4.01)
PADI6	Chr 1:17714971	G>A	rs74834315	0.0203126	0.0014096	0	Yes	V259I	Uncertain significance	Benign (0.003)	Deleterious (0.05)	Destabilizing (-0.88)
NLRP5	Chr 19:56539284	G>A	rs34175666	0.00494142	0.00742482	0	No	R562H	Uncertain significance	Possibly damaging (0.54)	Deleterious (0.02)	Destabilizing (-0.13)
NLRP5	Chr 19:56545075	G>A	rs768443657	4.06E-06	8.95E-06	0	Yes	R872K	Uncertain significance	Possibly damaging (0.761)	Deleterious (0.05)	Destabilizing (-0.89)
NLRP5	Chr 19:56569629	C>G	rs12462795	0.16983	0.145242	0.02203	Yes	S1108C	Benign	Probably damaging (0.976)	Deleterious (0)	Stabilizing (0.65)
NLRP5	Chr 19:56572875	G>A	rs36118060	0.169579	0.144598	0.02177	Yes	R1195Q	Benign	Benign (0.293)	Tolerated (0.11)	Destabilizing (-1.02)
NLRP4	Chr 19:56370038	G>A	rs111284755	0.00375796	0.00581119	2.00E-05	Yes	A427T	Uncertain significance	Benign (0.044)	Tolerated (0.22)	Destabilizing (-0.11)
NLRP2	Chr 19:55493728	C>T	rs17699678	0.0884999	0.110534	0.0121	Yes	T221M	Benign	Possibly damaging (0.672)	Deleterious (0.04)	Stabilizing (0.21)
NLRP2	Chr 19:55494121	T>G	rs147585490	0.00123848	0.00186774	0	Yes	I352S	Uncertain significance	Possibly damaging (0.707)	Deleterious (0)	Destabilizing (-1.52)
NLRP2	Chr 19:55494126	A>G	rs61735077	0.00506446	0.00789988	5.00E-05	Yes	I354V	Uncertain significance	Benign (0.02)	Tolerated (0.41)	Destabilizing (-1.93)
NLRP2	Chr 19:55494157	G>A	rs4306647	0.101262	0.0407483	0.00153	Yes	R364K	Uncertain significance	Possibly damaging (0.487)	Tolerated (0.22)	Destabilizing (-0.65)

NLRP2	Chr 19:55494747	G>A	rs61735086	0.00185715	9.67E-05	0	Yes	A561T	Uncertain significance	Benign (0.055)	Tolerated (0.32)	Destabilizing (-1.02)
**NLRP2*	Chr 19:55493651	C>T	rs10403648	0.102921	0.0419425	0.00181	Yes	F195F	Uncertain significance	-	-	-
**NLRP2*	Chr 19:55494233	G>A	rs759650015	7.58E-05	6.18E-05	0	Yes	A389A	Uncertain significance	-	-	-
***NLRP2*	Chr 19:55481394	C>T	rs142463014	0.00906567	0.00939562	0.00014	Yes	S4L	Uncertain significance	Benign (0.129)	Tolerated (0.09)	Stabilizing (0.62)
***NLRP7*	Chr 19:55439115	A>G	rs141473720	1.99E-05	1.76E-05	0	-	Y947H	Uncertain significance	Benign (0.009)	Tolerated (0.25)	-

Allelic and homozygous genotype frequencies were obtained querying the gnomAD- Genome database v2.1.1 with the tabix command-line utility. [a] Variant frequencies in all populations, [b] variant frequencies in non-Finnish European population, [c] frequencies of the variant homozygous genotype in non-Finnish European (NFE) population. [d] The ACMG criteria for classifying pathogenicity of the variants are reported by Richards et al., 2015. *Two synonymous variants identified in heterozygosity in proband's mother of family 10. ** Rare predicted not damaging variant identified in heterozygosity in mothers of family 8 (*NLRP2* - rs142463014) and family 6 (*NLRP7* - rs141473720).
